# Sclerostin and bone remodeling biomarkers responses to whole-body cryotherapy (− 110 °C) in healthy young men with different physical fitness levels

**DOI:** 10.1038/s41598-021-95492-8

**Published:** 2021-08-09

**Authors:** Anna Straburzyńska-Lupa, Tomasz Cisoń, Marta Gomarasca, Anna Babińska, Giuseppe Banfi, Giovanni Lombardi, Ewa Śliwicka

**Affiliations:** 1 Department of Physical Therapy and Sports Recovery, Poznan University of Physical Education, Poznań, Poland; 2 Department of Physiotherapy, State University of Applied Science in Nowy Sącz, Nowy Sącz, Poland; 3 Laboratory of Experimental Biochemistry and Molecular Biology, IRCCS Istituto Ortopedico Galeazzi, Milan, Italy; 4 Department of Endocrinology and Internal Medicine, Medical University of Gdansk, Gdańsk, Poland; 5 Vita-Salute San Raffaele University, Milan, Italy; 6 Department of Athletics, Strength and Conditioning, Poznan University of Physical Education, Poznań, Poland; 7 Department of Physiology and Biochemistry, Poznan University of Physical Education, Królowej Jadwigi Str. 27/39, 61-871 Poznań, Poland

**Keywords:** Biochemistry, Bone

## Abstract

We investigated the effects of single and repeated exposures to whole-body cryotherapy on biomarkers of bone remodeling and osteo-immune crosstalk: sclerostin, osteocalcin (OC), C-terminal cross-linked telopeptide of type I collagen (CTx-I), osteoprotegerin (OPG) and free soluble receptor activator for nuclear factor κ B ligand (sRANKL). The study included 22 healthy males, grouped in high physical fitness level (HPhL) and low physical fitness level (LPhL), all undergone 10 consecutive sessions in a cryogenic chamber (− 110 °C). We observed a significant time-effect on sclerostin (*p* < 0.05), OC (*p* < 0.01), CTx-I (*p* < 0.001), OC/CTx-I (*p* < 0.05), and significant differences in sRANKL between the groups (*p* < 0.05) after the 1st cryostimulation; a significant time-effect on OC (*p* < 0.001) and OC/CTx-I (*p* < 0.001) after the 10th cryostimulation, and a significant time-effect on CTx-I (*p* < 0.001) and OC/CTx-I (*p* < 0.01) after 10 sessions of WBC. In conclusion, in young men, the first exposure to extreme cold induced significant changes in serum sclerostin. The changes in sRANKL, between groups, suggest that fitness level may modify the body's response to cold. The effects of the first stimulus and the whole session are not identical, probably due to the physiological development of habituation to cold.

Whole body cryotherapy (WBC), also referred to as whole-body cryostimulation^1^, is a procedure based on short exposures (1–3 min) to extremely cold air (− 110 °C) in a controlled chamber^2^. It is used especially for its analgesic and anti-inflammatory effects in rheumatic diseases^3–6^, as well as a preventative strategy against the consequences of inflammation and pain caused by exercise^1^. During cold exposure, the sympathetic tone is increased and many homeostatic responses are activated in order to minimise heat loss (vasoconstriction) and to increase heat production (shivering, and non-shivering thermogenesis in brown adipose tissue)^7,8^.

The results of the conducted studies suggest that energy and bone metabolism are interacting with each other^9–12^. Bone turnover is strikingly dependent upon energy availability/demand and is regulated by several energy-related hormones, myokines, adipokines^12^, and neurotransmitters (i.e. insulin, leptin, adiponectin, epinephrine/norepinephrine)^13^. A way by which bone interacts with energy metabolism, is attributed to osteocalcin (OC) which acts on pancreatic cells and adipocytes, at least in rodents and in vitro models^12^. Thus, according to this new vision, glucose homeostasis, fat metabolism, adipose tissue metabolism, and bone remodeling are closely linked and regulate one the other in order to maintain the energy homeostasis^14,15^. Moreover, myokines participate in the autocrine regulation of metabolism, as well as in the endocrine regulation of other tissues and organs (including bone, liver, and adipose tissue)^16^. Recently, our studies, conducted in the young healthy men, have shown significant acute changes of irisin and myostatin among subjects with low cardiorespiratory levels after the tenth WBC exposure^17^.

Sclerostin, a Wnt signaling pathway antagonist^18^, is a glycoprotein primarily secreted by osteocytes^19^. This osteokine downregulates bone formation and promotes bone resorption by increasing expression and release of receptor activator of nuclear factor κB ligand (RANKL) from osteoblasts^20^. It has been suggested that muscle shivering, although within small range, might also affect bone metabolism^21^. Sclerostin may also have non-skeletal effects, since the pleiotropic involvement of the Wnt signalling pathway, i.e., adipocyte differentiation and fat metabolism^18^, as well as myoblast proliferation and differentiation^22^, and modulation of neural circuit functioning^13^. Its expression is regulated by a complex, not fully understood, mechanisms involving mechanosensing and systemic hormones (such as glucocorticoids, parathyroid hormone), cytokines, vitamin D, glucose^18,23^.

The RANKL/RANK/OPG system, a key homeostatic axis linking bone metabolism and immune function, participates in the regulation of bone resorption. RANKL, that is expressed by osteoblasts and immune cells, activates its receptor RANK expressed on the surface of osteoclasts and osteoclast precursors and, thus, it induces osteoclastogenesis and stimulates the bone resorption-related osteoclast function. OPG, instead, is a soluble decoy receptor that, by binding with soluble RANKL, prevents the activation of RANK and, hence, it protects bone from excessive resorption. Therefore, the RANKL-to-OPG ratio is a reliable indicator of the bone metabolic status^24^. Further, OPG, other than an inhibitor of bone resorption^24^, is a target gene of the Wnt/β-catenin signaling^21^. The activation of Wnt/β-catenin pathway in osteoblasts increases the expression of OPG and reduces bone resorption. Because sclerostin antagonizes the Wnt/β-catenin signal pathway, changes in sclerostin expression can also modulate resorption by regulating OPG^25^.

OC, it is a γ-carboxyglutamic acid protein expressed and secreted almost exclusively by osteoblasts^26^. Fully carboxylated OC (c-OC) binds to hydroxyapatite (HAP) in bone extracellular matrix, and allows the correct growth of the HAP crystals^26^. On the other side, un(der)carboxylated (uc-OC) osteocalcin seems to play a role in glucose and energy metabolism via pancreatic β-cells and adipocytes, although it has been demonstrated only in rodent models^27^. Still, the specific extra-skeletal functions of OC and its differently carboxylated forms remain unclear.

It should be noted that, despite the increasing number of studies on the effects of WBC, only few of them concern bones and none of them have focused on sclerostin. Some previous studies suggested that physical fitness level might modify the effect of cryotherapy^17,28^. A study by Maeda^29^ suggests that, probably due to a greater capacity for peripheral vasoconstriction in response to cold, there is less heat lost in fitted individuals. Also, physical fitness is associated with a greater ability to maintain metabolic heat production in a cold environment at resting metabolic level of a thermoneutral condition^29^.

Due to the involvement of skeletal metabolism in the management of the energy substrates and, hence, in thermogenesis, we investigated the effects of single and repeated WBC exposures in physically active volunteers with different physical fitness levels on the circulating levels of biomarkers of bone remodeling and bone-energy metabolism and, particularly, sclerostin, OPG, OC, CTx-I and sRANKL. Taking into account that factors, such as sports participation or gender can affect sclerostin levels^11,30^, the study was conducted only among young non-training male. It also should be noted that men respond to cold stress by increasing their metabolism instead to cool skin temperatures^31^.

## Results

### Pre-treatment data

An overview of baseline characteristics in HPhL and LPhL group are given in Tables 1 and 2. Participants` demographics, excluding maximal oxygen consumption (VO_2_ max) levels (*p* < 0.001)^17^, total BMD (*p* < 0.05) and lumbar spine BMD (*p* < 0.05) were comparable between the two groups. No significant differences in biochemical variables were found between the groups, except for sclerostin (*p* < 0.01). The mean values of sclerostin in LPhL group were not homogeneous, with a quite high SD indicating a quite large inter-individual variability.

**Table 1:** Pre-therapy bone mineral density parameters of young healthy men.

	HPhL group	LPhL group	*p*-value
**variables described in our previous study**^17^:			
Body height [cm]	180.09 ± 4.68	182.31 ± 5.09	0.298
Body mass [kg]	76.18 ± 5.47	74.11 ± 6.64	0.434
BMI [kg/m^2^]	23.50 ± 1.59	22.34 ± 2.33	0.187
VO_2_ max [ml kg^-1^ ·min^-1^]	49.86 ± 4.74	38.59 ± 3.01	< 0.001
Total BMD [g/cm^2^]	1.349 ± 0.113	1.249 ± 0.085	0.0312
Total BMD Z-score	1.456 ± 1.373	0.556 ± 0.794	0.1080
Total BMC [g]	3007.89 ± 1001.50	2881.65 ± 981.41	0.1310
Total femur BMD [g/cm^2^]	1.304 ± 0.169	1.185 ± 0.161	0.1147
Total femur BMD Z-score	1.533 ± 1.515	0.538 ± 1.103	0.1357
Femoral neck BMD [g/cm^2^]	1.317 ± 0.168	1.194 ± 0.173	0.1070
Femoral neck BMD Z-score	1.589 ± 1.440	0.722 ± 1.273	0.1942
Lumbar spine BMD [g/cm^2^]	1.265 ± 0.114	1.144 ± 0.093	0.0128
Lumbar spine BMD Z-score	1.256 ± 1.154	0.278 ± 0.907	0.0630

Data are presented as mean ± SD. *BMI* body mass index, *BMD* bone mineral density, *BMC* bone mineral content.

**Table 2:** Pre-therapy biochemical indices in young healthy men.

	HPhL group	LPhL group	*p*-value
Sclerostin [pmol/mL]	33.01 ± 5.76	29.65 ± 14.73	0.0065
OC [ng/mL]	14.51 ± 3.70	14.22 ± 1.86	0.8225
CTx-I [ng/mL]	1.10 ± 0.45	1.08 ± 0.46	0.9660
OC/CTx-I	15.21 ± 6.98	16.14 ± 9.49	0.9476
OPG [pmol/mL]	3.54 ± 2.06	4.36 ± 2.75	0.3933
sRANKL [pmol/mL]	0.23 ± 0.12	0.19 ± 0.07	0.4701
OPG/sRANKL	19.68 ± 14.13	27.43 ± 16.23	0.2122

Data are presented as mean ± SD. *OC* osteocalcin; *CTx-I* C-terminal cross-linked telopeptide of type I collagen, *OPG* osteoprotegerin, *sRANKL* free soluble receptor activator for nuclear factor κ B ligand.

### Effect of single cold stimulations (first and tenth exposures)

**Figure 1:**
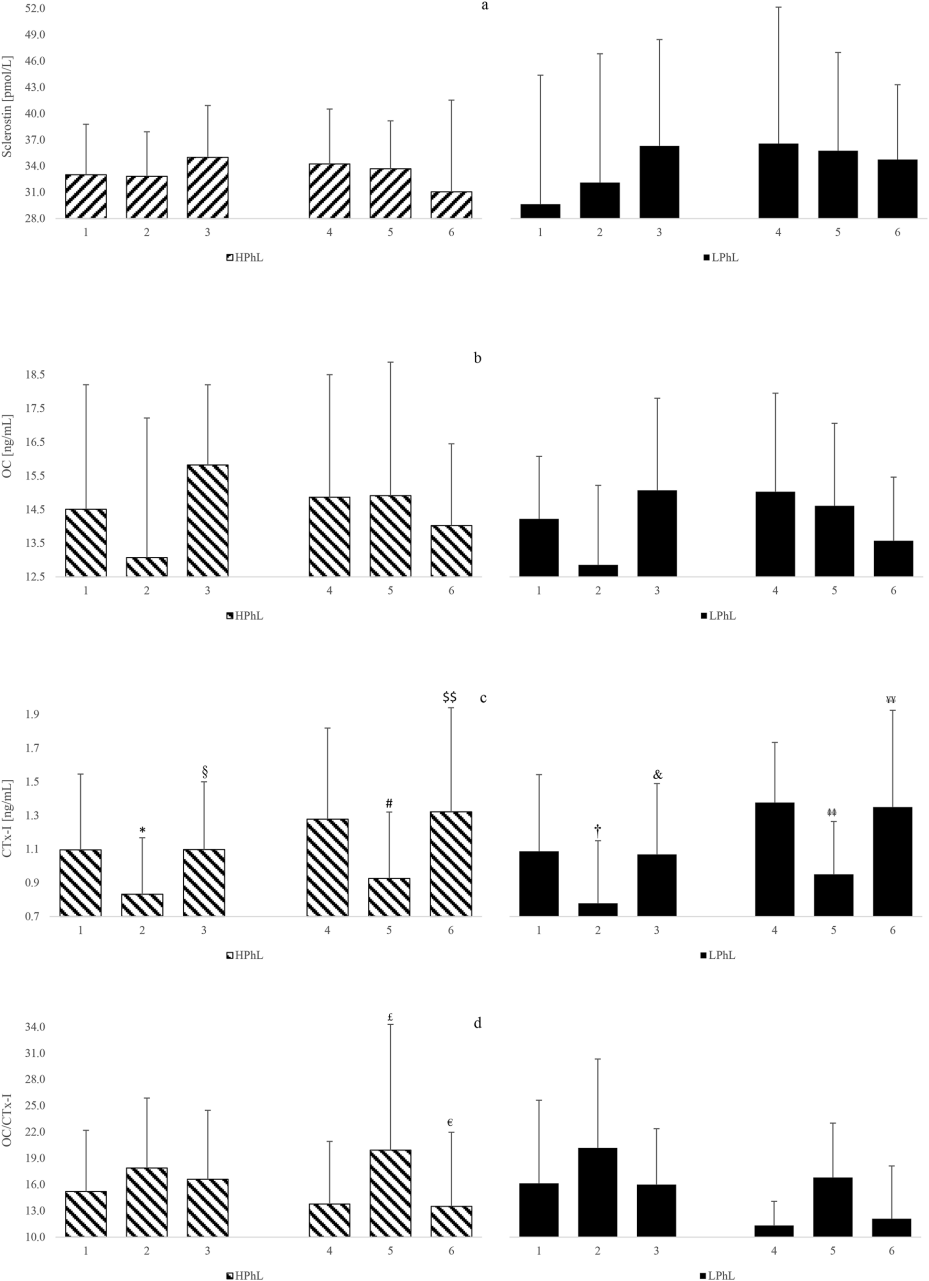
Blood concentrations of sclerostin, OC, CTx-I, OC/CTx-I, OPG, sRANKL and OPG/sRANKL, in young healthy men (first and tenth cryostimulations) (**a**) sclerostin, (**b**) OC, (**c**) CTx-I, (**d**) OC/CTx-I. Data are presented as means. *p < 0.05 significant differences between measurements before (1) and 30 min (2) after the first cryostimulation in the HPhL group. ^§^*p* < 0.05 significant differences between measurements 30 min (2) and 24 h (3) after the first cryostimulation in the HPhL group. ^†^*p* < 0.05 significant differences between measurements before (1) and 30 min (2) after the first cryostimulation in the LPhL group. ^&^*p* < 0.05 significant differences between measurements 30 min (2) and 24 h (2) after the first cryostimulation in the LPhL group. ^#^*p* < 0.05 significant differences between measurements before (4) and 30 min (5) after the tenth cryostimulation in the HPhL group. ^$$^*p* < 0.01 significant differences between measurements 30 min (5) and 24 h (6) after the tenth cryostimulation in the HPhL group. *p* < 0.01 significant differences between measurements before (4) and 30 min (5) after the tenth cryostimulation in the LPhL group. ^¥¥^*p* < 0.01 significant differences between measurements 30 min (5) and 24 h (5) after the tenth cryostimulation in the LPhL group. ^£^*p* < 0.05 significant differences between measurements before (4) and 30 min (5) after the tenth cryostimulation in the HPhL group. ^€^*p* < 0.05 significant differences between measurements 30 min (5) and 24 h (6) after the tenth cryostimulation in the HPhL group.

**Figure 2:**
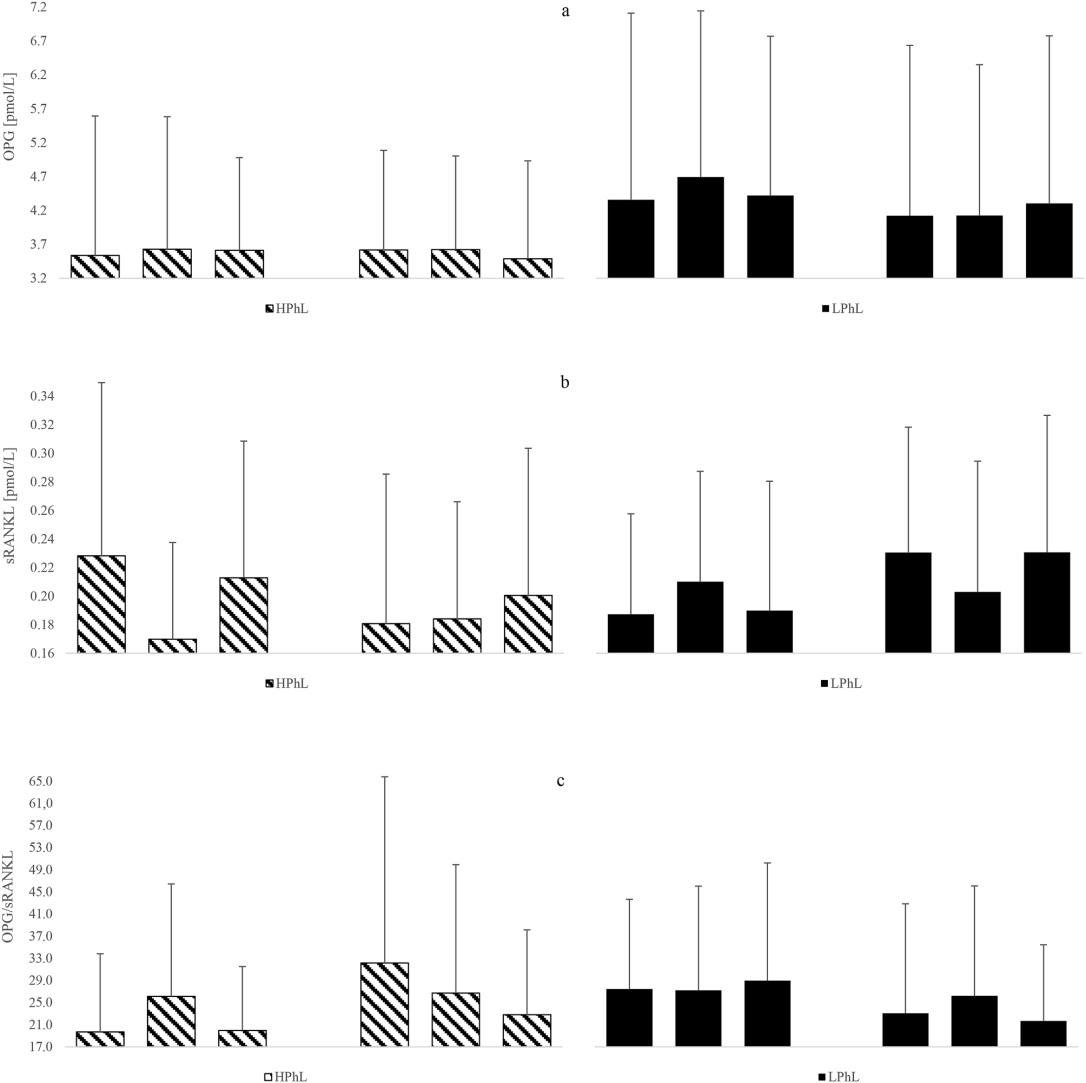
Blood concentrations of OPG, sRANKL and OPG/sRANKL in young healthy men (first and tenth cryostimulations). (**a**) OPG, (**b**) sRANKL, (**c**) OPG/sRANKL. Data are presented as means.

The effects of the acute cold stimulation at either the beginning (1st session) or the end (10th session) of the WBC treatment on blood concentration of sclerostin, OC, CTx-I, OC/CTx-I ratio, OPG, sRANKL, OPG/sRANKL ratio are presented in Figs. 1 and 2.

During the 1st cryostimulation, significant time-effects on sclerostin (*p* < 0.05), OC (*p* < 0.01), CTx-I (*p* < 0.001) and OC/CTx-I (*p* < 0.05) were noted. Both groups experienced a significant decrease of CTx-I concentrations after the acute cold stimulation, immediately after the 1st (HPhL, *p* < 0.05; LPhL, *p* < 0.05), when compared to the pre-stimulus level and a significant increase 24 h after the 1st exposure (HPhL (*p* < 0.05; LPhL, *p* < 0.05), when compared to the level immediately after the stimulation (Fig. 1c). In case of sRANKL, a repeated-measures ANOVA revealed a Group × Time interaction, which suggested that there was a differential effect between the groups following 1st exposure to cold (*p* < 0.05) (Table 3).

**Table 3:** Summary ANOVA results on effects of single (1st and 10th) and 10WBC sessions.

	1st WBC session			10th WBC session			10 sessions of WBC		
	F	*p*	ƞ^2^	F	*p*	ƞ^2^	F	*p*	ƞ^2^
**Sclerostin**									
Group	0.0518	0.8223	0.0026	0.4819	0.4956	0.0235	0.0458	0.8327	0.0023
Time	3.2518	0.0491	0.1399	1.1765	0.3188	0.0556	1.9117	0.1611	0.0872
Group × time	0.8911	0.4182	0.0427	0.1348	0.8742	0.0067	2.2716	0.1163	0.1020
**OC**									
Group	0.1618	0.6918	0.0080	0.0299	0.8644	0.0015	0.1028	0.7518	0.0051
Time	7.8947	0.0013	0.2830	3.2198	0.0505	0.1387	2.1040	0.1353	0.0952
Group × time	0.1096	0.8965	0.0054	0.2202	0.8033	0.0109	0.0190	0.9812	0.0009
**CTx-I**									
Group	0.0367	0.8499	0.0018	0.0734	0.7892	0.0037	0.0065	0.9367	0.0003
Time	14.9959	0.0000	0.4285	19.3975	0.0000	0.4924	13.9968	0.0000	0.4117
Group × time	0.0758	0.9271	0.0038	0.1652	0.8483	0.0082	0.0354	0.9653	0.0018
**OC/CTx-I**									
Group	0.0716	0.7918	0.0036	0.5200	0.4792	0.0253	0.1199	0.7328	0.0060
Time	4.7954	0.0136	0.1934	14.0839	0.0000	0.4132	7.4374	0.0018	0.2711
Group × time	0.7862	0.4625	0.0378	0.2434	0.7851	0.0120	0.9947	0.3788	0.0474
**OPG**									
Group	0.9866	0.3324	0.0470	0.5645	0.4612	0.0274	0.7017	0.4121	0.0339
Time	0.5144	0.6017	0.0251	0.0089	0.9912	0.0004	0.0425	0.9584	0.0021
Group × time	0.2240	0.8003	0.0111	0.3857	0.6825	0.0189	0.2584	0.7736	0.0128
**sRANKL**									
Group	0.0581	0.8119	0.0029	0.8418	0.3698	0.0404	0.0060	0.9390	0.0003
Time	0.5753	0.5671	0.0280	0.9702	0.3878	0.0463	0.7067	0.4993	0.0341
Group × time	3.2354	0.0498	0.1392	0.4845	0.6196	0.0237	2.0442	0.1428	0.0927
**OPG/sRANKL**									
Group	0.8052	0.3802	0.0387	0.2142	0.6485	0.0106	0.1100	0.7436	0.0055
Time	0.6098	0.5484	0.0296	0.7528	0.4776	0.0363	0.7165	0.4946	0.0346
Group × time	1.0825	0.3485	0.0513	0.5401	0.5869	0.0263	0.9478	0.3961	0.0452

*OC* osteocalcin, *CTx-I* C-terminal cross-linked telopeptide of type I collagen, *OPG* osteoprotegerin, *sRANKL* free soluble receptor activator for nuclear factor κ B ligand.

After the 10th cryostimulation, significant time-effects on OC (*p* < 0.001) and OC/CTx-I (*p* < 0.001) were noted. Both groups experienced a significant decrease of CTx-I concentrations after the acute cold stimulation, immediately after the 10th exposure (HPhL, *p* < 0.05; LPhL, *p* < 0.01), when compared to the pre-stimulus level and a significant increase 24 h after the 10th exposure (HPhL, *p* < 0.01; LPhL, *p* < 0.01), when compared to the level recorded immediately after stimulus (Fig. 1c). In the HPhL group there were a significant increase of OC/CTx-I after the acute cold stimulation, immediately after the 10th (*p* < 0.05), when compared to the pre-stimulus level, and a significant decrease 24 h after the 10th exposure (*p* < 0.05), when compared to the level immediately after stimulus (Fig. 1d).

### Effect of 10 WBC sessions

After 10 sessions of WBC, i.e., the complete cycle, significant time-effects on CTx-I (*p* < 0.001) and OC/CTx-I (*p* < 0.01) were noted. In both groups, it was noted a significant increase of CTx-I concentrations immediately after the 10th (HPhL, *p* < 0.01; LPhL, *p* < 0.01), when compared to the 1st pre-stimulus level, while, in HPhL group, CTx-I significantly increased also 24 h after the 10th exposure (*p* < 0.05), when compared to the level immediately after stimulus (Fig. 1c).

### Relationships between variables

In the analyses of our baseline data, we identified a significant positive correlation between serum sclerostin and myostatin, measured in our previous study^17^ (r = 0.42; *p* = 0.0495).

Table 4 presents the results of the correlation analysis for each group after the 1st and the 10th cryotherapy sessions in the changes (Δ) observed in biochemical indices before and 30 min after the treatment, as well as 24 h after cryostimulation. In the LPhL group, after the 1st WBC, changes (Δ_1–3_) in sclerostin were positively correlated with changes (Δ_1–3_) in OPG/sRANKL (r = 0.64; *p* = 0.0353), irisin (r = 0.65; *p* = 0.0289) and hsIL-6 (r = 0.65; p = 0.0289). We also found positive relationships between changes (Δ_1–5_) in the levels of sclerostin and OC (r = 0.73; *p* = 0.0112), as well as sclerostin and irisin (r = 0.85; *p* = 0.0008). The changes (Δ_1–6_) in the levels of sclerostin were positively correlated with CTx-I in LPhL group (r = 0.77; *p* = 0.0053) and with myostatin in HPhL group (r = 0.67; *p* = 0.0233).

**Table 4:** Spearman's rank correlation coefficients of tested variables.

Variables	HPhL group		LPhL group	
	r	*p-*value	r	*p-*value
Δ_1–3_ Sclerostin/Δ_1–3_ OPG/sRANKL			0.64	0.0353
Δ_1–5_ Sclerostin/Δ_1–5_ OC			0.73	0.0112
Δ_1–6_ Sclerostin/_1–6_ sRANKL/			0.75	0.0085
Δ_1–6_ Sclerostin/Δ_1–6_ CTx-I			0.77	0.0053
**Correlations with variables described in our previous study**^17^:				
Δ_1–3_ Sclerostin/Δ_1–3_ Irisin/			0.65	0.0289
Δ_1–3_ Sclerostin/Δ_1–3_ hsIL-6/			0.65	0.0289
Δ_1–5_ Sclerostin/Δ_1–5_ Irisin			0.85	0.0008
Δ_1–6_ Sclerostin/Δ_1–6_ Myostatin	0.67	0.0233		

1 = before first WBC, 2 = 30 min after first WBC, 3 = 24 h after first WBC, 4 = before tenth WBC, 5 = 30 min after tenth WBC, and 6 = 24 h after tenth WBC.

## Discussion

To the best of our knowledge, this is the first study assessing the effect of acute and whole-cycle WBC treatment on sclerostin levels in healthy young men with different physical fitness levels. At the same time, demonstrated changes in sRANKL levels seem to confirm the results obtained in our previous study^17^, indicating that an extreme cold stimulus may modify the body's response depending on the physical fitness level.

In a preliminary investigation, we observed significantly higher concentration of sclerostin, and higher total and lumbar spine BMD, in the HPhL group as compared to the LPhL group. Zagrodna et al.^30^ suggested that the circulating sclerostin concentration can reflect the total weight of the skeleton—a larger skeleton can produce and release more sclerostin for circulation.

The findings of the present study showed significant time–effects and, thus, possibly the effects of the intervention, regardless the physical activity level, on sclerostin levels 24 h after the first WBC exposure compared to the baseline. Its levels have increased by 23% in LPhL and only by 6% in the HPhL group, although without reaching the statistical significance. A comparable increase of sclerostin levels was observed after mechanical stimulation (about 40 min resistance exercise or walking) by Gombos et al.^32^. It should be noted that studies carried out in recent years indicate that mostly long lasting mechanical stress can play a role in regulating sclerostin levels^33^ and, probably, acute exercise may exert this effect but only when long-lasting and high-intensity^34,35^. However, recent studies have shown that circulating sclerostin levels may be altered, not only in response to systemic mechanical stimuli, but also by hormonal stimuli in various physiological and pathophysiological conditions^23^. In our study, in order to minimize the effect of exercise on the regulation of bone markers, following each WBC, the participants performed a short physical exercise on a cycloergometer^36^.

The acute increase of sclerostin after a single WBC application is of particular interest, although the biological meaning cannot be evinced from the current setting. However, it is possible to hypothesize a role of the WBC-dependent sympathetic activation. Indeed, osteocytes, the main cell source for sclerostin, express β-adrenergic receptors and their activation is associated, in vivo, to enhanced osteoclastogenesis^13^. This knowledge somehow matches with our findings since, significant positive relationship between changes (Δ_1–6_) in the concentration of sclerostin and CTx-I in LPhL group, which marks an enhanced osteoclastic activity. Further, sclerostin raised by a higher value in LPhL subjects who are possibly more sensitive to the adrenergic stimulation than subjects accustomed to physical activity^37^. It is also in line with other studies that showed that physically fit individuals have more effective thermoregulatory capacity against cold stress than physically unfit individuals^38^. However, to our knowledge, a demonstration about a direct effect of adrenergic stimulation on sclerostin expression/release still lacks.

Both groups experienced a significant decrease of CTx-I concentrations after the acute cold stimulation, immediately after the 1st session, when compared to the pre-stimulus level, and a significant increase 24 h after the 1st exposure, when compared to the level immediately after stimulus. On the one hand, this may indicate that CTx-I is a very sensitive marker in response to a single cold stimulation. On the other hand, however, the observed increase in this bone resorption marker 24 h after the end of the entire treatment (by 20% in HPhL and 25% in LPhL) may suggests a long-lasting modulation of the resorption-to-formation balance in bone. Similar findings have been presented in other studies with respect to CTx-I and acute exercise^34,39,40^. Whereas, as reported by others authors^41^, markers of osteogenesis are more sensitive than markers of bone resorption in response to exercise.

The greater increase in sclerostin levels noted by us in LPhL group after therapy and the significant positive relationship between changes (Δ_1–6_) in the concentration of sclerostin and those of RANKL and the bone resorption marker CTx-I, confirms the negative effect of cold exposure on bone in these subjects. Additionally, we observed a positive correlation between changes (Δ_1–5_) in the concentration of sclerostin and those of the formation marker OC. This is consistent with Gombos et al.^32^, who stated that immediately after stimulus (mechanical loading) begins bone resorption and at the same time, only slightly less, bone formation also begins.

We also observed a significant time–effect, with the 1st stimulus of WBC, on serum OC concentrations. In both groups, indeed, we observed a slight decrease of OC concentrations after the first session (by 10% in HPhL and 9% in LPhL) followed by the increase in the subsequent 24 h after the 1st exposure, when compared to the pre-treatment levels (by 9% in HPhL and 6% in LPhL). There were no significant differences in OC level after completed therapy when compared to baseline values. Some authors point out that the circadian alterations of OC may be inverse to the changes in cortisol concentration^42^. Leppäluoto et al.^43^ observed in healthy females a slight increase of plasma cortisol levels after first cold exposure (winter swimming and whole-body cryotherapy) and significant decrease in 4–12 weeks of repetitive procedures. These results were probably related to the habituation reaction.

The results of studies conducted on recreationally active volunteers showed that a single 3-min cryostimulation is able to induce a strong autonomic response, as expressed by the increased sympathetic activation (i.e., increased plasma norepinephrine and systolic/diastolic blood pressure), and increased parasympathetic control of heart rate^44^. However, Luis et al.^45^ described a sort of adaptation to cold exposure since they observed a lower autonomic response after five daily exposures (− 110 °C), compared to the first.

Similar conclusions were reached by other authors who performed animal studies^9^, and stated that cold exposure negatively affects bone remodeling in mice. This is related to the increasing sympathetic tone through the activation of β_2_-adrenergic receptors, which stimulates bone resorption partly by increasing RANKL expression in osteoblasts and inhibits bone formation^9^.

In the current study, significant differences in sRANKL between both groups after first stimulus were noted. Also, although non- significant, a different response of sRANKL to the cold stimulus depending on the physical fitness 24 h after 10 sessions of WBC, compared to pre-therapy levels were observed: a slight increase (21%) in the LPhL, and a slight decrease (13%) in the HPhL group. These results are consistent with those reported by Galliera et al.^25^ in elite rugby players. The authors observed that 5 WBC sessions had no effects on RANKL, although, in contrast in our present ones they evidenced a significant increase in OPG. Such a different behaviour in OPG response may have been due to the fact that professional rugby players were submitted to regular training regimen that may be accounted as a weight-bearing activity stimulating bone anabolism.

Galliera et al.^25^ also reported that, after 5 WBC sessions, the OPG-to-RANKL ratio (the resorption-to-formation balance index), significantly increased, which may indicate an osteogenic effect of WBC when associated with adequate training. In our study, we observed a slight transient increase of this ratio only in the HPhL group: by 36% immediately after the 10th session and still by 16% 24 h after exposure while, in the LPhL group, we recorded a slight decrease: by 4% immediately after the 10th to 21% 24 h later the last exposure, compared to the pre-therapy level. This suggests an adverse effect of WBC on bone in low physical activity level subjects. Overall, our results confirm the findings from other previous studies^11,17^ which showed that a WBC session contributed to diverse systemic thermogenic responses depending on the level of physical fitness and this in turn have a variety effects on skeletal metabolism. OPG, is not only a marker of bone formation, but it is also involved in the modulation of the bony effect of inflammation by interfering with the RANK/RANKL signaling pathway^46^. Further, the OPG/RANKL system plays also important role in muscle inflammation^47^. It seems that the demonstrated differences depending on the physical level may indicate developing adaptation to reduce inflammation in more active people.

We suppose that increased sclerostin levels may be also associated with inflammatory response which confirms the significant positive relationship between changes (Δ_1–3_) in sclerostin concentration and hsIL-6 (measured in our previous study^17^) and OPG/sRANKL ratio observed after the first procedure in LPhL group.

The close relationship between skeletal muscle and bone functions has been established in the literature^15,48^. Recently, our studies conducted in the same group of participants have shown that exposure to extreme cold in LPhL group LPhL significant increased irisin 24 h after the tenth stimulus in compare to pre-stimulus level, and myostatin 24 h after the tenth stimulus in compare to 30 min after that stimulus^17^. Because irisin and myostatin also participate in the control of energy balance, cryostimulation can lead to changes in their concentration^17^ which may also affect the bone metabolism. Kim et al.^49^ suggest that irisin through its integrin receptor promotes bone resorption by increasing sclerostin expression in osteocytes. In the analyses of our baseline data, we identified a significant positive correlation between serum sclerostin and myostatin, which supports hypothesis of a biochemical cross-talk between the bone and muscle tissue^22^. However, we have not found relationship between sclerostin and irisin concentrations. These results are contrary to the data published by Klangjareonchai et al.^50^, who demonstrated that serum sclerostin is highly correlated with serum irisin in adults with prediabetes.

At the same time, we found a positive correlation between changes (Δ_1–6_) in the concentration of sclerostin and those of myostatin in HPhL group and between changes (Δ_1–3_; Δ_1–5_) in the concentration of sclerostin and those of irisin in LPhL group. Fournier et al.^51^ in the study on animal models showed that blocking the myostatin receptor evoked an elevation of BAT, an improvement of its mitochondrial function, resulting in better cold tolerance, and enhanced energy expenditure. Increased energy expenditure occurs in the cold, not in thermoneutrality, where non-shivering thermogenesis is minimal. On the other hand, Acosta et al.^52^ showed that intensity levels of physical activity were neither associated with BAT volume/activity nor with the skeletal muscles activity. Thus, the results of our research suggested that skeletal metabolism is affected by muscle function in response to cold exposure, however the mechanism remains to be further studied.

Our study has some potential limitations. In particular, a small number of subjects participated in this study. Additionally, the study period was only to 24 h after ten application, possibly too short to observe the long-term effects of WBC. The strength of the present study, instead, was that it examined the impact of both: single sessions (the first and the tenth ) and series of 10 sessions on the circulating levels of biomarkers of bone remodeling and bone-energy metabolism and, particularly, sclerostin, OPG, OC, CTx-I and sRANKL. Some researches indicate that bone turnover markers, like CTx-I and OC, are influenced by diurnal changes^53,54^. In addition, it is indicated that other sources of variability should also be taken into account when measuring CTx-I, like nutrition, day-to-day variability, seasonal variation, but as it seems not acute exercise. Although efforts have been made to take these factors into account, it cannot be excluded that the noted CTx-I changes are not biologically significant^55^. At the same time the lack of a control group and control conditions in our study, makes it difficult, especially for CTx-I, to distinguish between the treatment effect and the diurnal variation. We also did not monitored the subjects’ diet, however, males taking any medications or dietary supplements that may affect bone health (e.g., vitamin D) were excluded from the study. In order to control this aspects, during the experiment, all participants were instructed not to change any aspect of their habits, such as diet, and avoid any form of exercise. Another potential limitation is that we have not calculated the changes in plasma volume that may have occurred with exposure to cold and exercise.

In conclusion, in young men, the first exposure to extreme cold induced significant changes in serum sclerostin. The changes in sRANKL between groups suggest that fitness level may modify the body's response to cold. The effects of the first stimulus and the whole session on some parameters are not identical, probably due to the development of physiological cold habituation.

## Methods

### Study group

This study involves further analysis of blood samples previously collected and partially analyzed^17^ in young men after WBC. The study involved a total of 26 male recreationally active volunteers. All study participants (aged 19–23) were in good state of physical health, free of injuries or chronic conditions, non-smokers, and not taking any medications or dietary supplements that may affect bone health (e.g., vitamin D) and not practicing any professional sport in the past. They did not present any contraindications to whole-body cold exposure. All of them had not previously undergone systemic cryotherapy.

In total, 22 subjects (mean age: 21 ± 1.17 years) completed the entire study protocol and were included in the analysis (4 participants did not finish the experiment due to personal reasons). The participants were divided into 2 groups based on the physical level (PhL) either high (HPhL) or low (LPhL) and physical activity status: group 1, HPhL with a higher VO_2_ max ≥ 43 ml min^−1^ kg^−1^ (n = 11), physical active ≥ 5 h/week^56^; and group 2, LPhL with a lower VO_2_ max < 43 ml min^−1^ kg^−1^ (n = 11), physical active ≤ 4 h/week. The characteristics of both groups are shown in Table 1.

**Figure 3:**
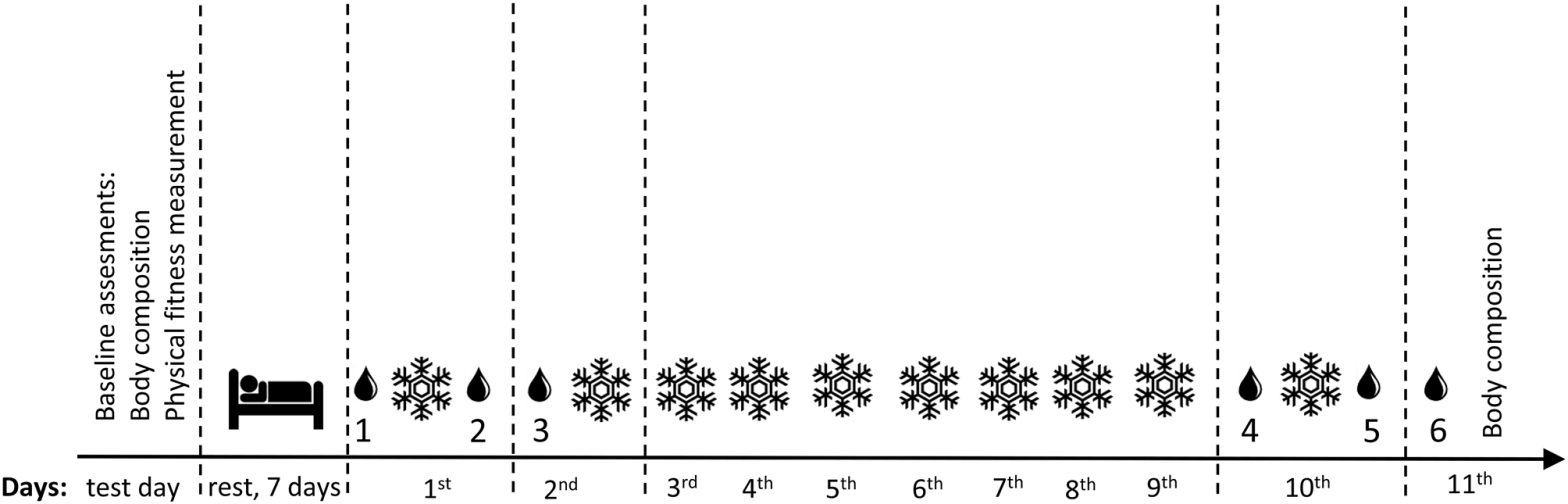
Experiment schedule.  = whole-body cryotherapy.  = blood collection. 1 = before first WBC, 2 = 30 min after first WBC, 3 = 24 h after first WBC, 4 = before tenth WBC, 5 = 30 min after tenth WBC, and 6 = 24 h after tenth WBC.

This study was conducted over 10 consecutive days in June. The research scheme is presented in Fig. 3. Each participant was allowed to familiarize with all testing procedures and provided a written informed consent prior to the study. The study protocol was approved by the Ethics Committee for Human Research at the Gdansk University of Medical Sciences (approval no. KB 28/17) and was performed in accordance with the Declaration of Helsinki.

### Anthropometric, body composition, and bone densitometry measurements

Body mass and body height were measured using a certified medical digital scale WB-3000 (TANITA Corporation, Tokyo, Japan), with an accuracy of 0.01 kg, and a mechanical measuring rod for body height HR-001 (TANITA Corporation, Tokyo, Japan), with an accuracy of 0.5 cm.

Dual-energy X-ray absorptiometry (DXA) body composition and bone mineral density (BMD) measurements were performed on an empty stomach using a GE Lunar Prodigy Primo Full Densitometer with the enCore Body Composition option (GE Healthcare Technologies, USA). The assessment of BMD was performed total body, left hip (total femur and femoral neck), and lumbar spine. All scans were acquired by the same technician. The short-term reproducibility of the applied DXA device has been shown to be 2.1% for total body, 1.1% for lumbar spine and 1.6% for femoral neck.

### Physical fitness measurement

One week before the start of the experiment, participants performed a treadmill exercise test, as described previously^17^. The circulation and respiratory parameters were monitored continuously using an ergospirometer (VO_2_ max Finder; MES, Poland). The heart rate was recorded every 5 s using a Polar Accurex Plus device (Polar Elektro, Finland).

Study participants were instructed not to perform any intense physical exercise in the 48 h preceding the procedures that could lead to dehydration and delayed onset muscle soreness (DOMS), and not to change any aspect of their habits (such as diet, and to avoid any form of exercise) during the experiment.

### Whole-body cryostimulation

All participants underwent a series of 3 min exposures to cold (once a day between 8:00–10:00 a.m.) for a total of 10 sessions in a cryogenic chamber (Zimmer Ice Lab, − 110 °C, Medizin System GmbH; Germany) as described previously^17^.

Following WBC, the participants performed physical exercise at 100 W on a cycloergometer (Keiser M3, Germany) for 15 min in order to allow the temperature recovery and to prevent shivering.

### Biochemical analyses

Blood samples for biochemical analyses were taken 6 times from the antecubital vein: before, 30 min after, and 24 h after the first exposure; and before the final cryotherapy session and 30 min and 24 h after recovery following the final cryotherapy session. The samples were collected with all safety standards into serum-separating tubes (9 mL, S-Monovette, SARSTEDT, Nümbrecht, Germany) and centrifuged at 2000×g for 10 min at 4 °C. The serum was separated from the sample and stored at − 70 °C.

OPG, free soluble high-sensitivity sRANKL and sclerostin serum concentrations were determined by commercially available immunoenzymatic assays (Biomedica GmbH & Co KG, Wien, Austria; test sensitivity: 0.07 pmol/L, 0.01 pmol/L and 3.2 pmol/L, respectively; intra-assay coefficients of variations (CV): 2.5%, 3%, 6.0%, respectively; inter-assay precision CV: 4.0%, 5%, 6.5%, respectively), as well as the levels of the bone resorption marker CTx-I (Immunodiagnostic Systems, Boldon, UK; test sensitivity: 0.02 ng/mL; intra-assay CV: 3%; inter-assay CV: 10.9%) and the bone formation marker OC (Quidel, San Diego, CA, USA; test sensitivity: 0.45 ng/mL; intra-assay CV: 10%; inter-assay CV: 9.8%).

### Statistical analysis

Data were presented as means and standard deviations (SD). The Shapiro–Wilk test was used to check the data for normality of distribution. Assumption on sphericity was tested using Mauchley’s test, verifying if variances of certain variables were identical and equal to respective co-variances. A two-way analysis of variance

(ANOVA) for repeated measures was used to analyze the differences in the biochemical indices between HPhL and LPhL groups. Bonferroni post hoc tests were performed to assess the significance of differences between pairs of measurements. Partial eta-squared (ƞ^2^) was calculated to determine the effect size. Relationships between variables were tested using Spearman’s rank correlation. A *p*-value < 0.05 was considered significant. Statistical analyses were performed using the Statistica 13.3 software package (TIBCO Software Inc., Palo Alto, CA, USA).

## Supplementary Information

Supplementary Information.

## Data Availability

The datasets generated during and/or analysed during the current study are available from the corresponding author on reasonable request.
